# Impact of immune thrombocytopenic purpura on clinical outcomes in patients with acute myocardial infarction

**DOI:** 10.1002/clc.23287

**Published:** 2019-11-11

**Authors:** Omar Chehab, Nadine Abdallah, Amjad Kanj, Mohit Pahuja, Oluwole Adegbala, Rami Z. Morsi, Tushar Mishra, Luis Afonso, Aiden Abidov

**Affiliations:** ^1^ Department of Internal Medicine Wayne State University Detroit Michigan; ^2^ Department of Internal Medicine Brigham and Women's Hospital Boston Massachusetts; ^3^ Cardiology Section, Department of Internal Medicine John D. Dingell VA Medical Center Detroit Michigan

**Keywords:** acute myocardial infarction, bleeding complications, coronary revascularization, immune thrombocytopenic purpura, thrombocytopenia, transfusion

## Abstract

**Background:**

Patients with immune thrombocytopenic purpura (ITP) admitted with acute myocardial infarction (AMI) may be challenging to manage given their increased risk of bleeding complications. There is limited evidence in the literature guiding appropriate interventions in this population. The objective of this study is to determine the difference in clinical outcomes in AMI patients with and without ITP.

**Methods:**

Using the United States national inpatient sample database, adults aged ≥18 years, who were hospitalized between 2007 and 2014 for AMI, were identified. Among those, patients with ITP were selected. A propensity‐matched cohort analysis was performed. The primary outcome was in‐hospital mortality. Secondary outcomes were coronary revascularization procedures, bleeding and cardiovascular complications, and length of stay (LOS).

**Results:**

The propensity‐matched cohort included 851 ITP and 851 non‐ITP hospitalizations for AMI. There was no difference in mortality between ITP and non‐ITP patients with AMI (6% vs7.3%, OR:0.81; 95% CI:0.55‐1.19; *P* = .3). When compared to non‐ITP patients, ITP patients with AMI underwent fewer revascularization procedures (40.9% vs 45.9%, OR:0.81; 95% CI:0.67‐0.98; *P* = .03), but had a higher use of bare metal stents (15.4% vs 11.3%, OR:1.43; 95% CI:1.08‐1.90; *P* = .01), increased risk of bleeding complications (OR:1.80; CI:1.36‐2.38; *P* < .0001) and increased length of hospital stay (6.14 vs 5.4 days; mean ratio: 1.14; CI:1.05‐1.23; *P* = .002). More cardiovascular complications were observed in patients requiring transfusions.

**Conclusions:**

Patients with ITP admitted for AMI had a similar in‐hospital mortality risk, but a significantly higher risk of bleeding complications and a longer LOS compared to those without ITP. Further studies are needed to assess optimal management strategies of AMI that minimize complications while improving outcomes in this population.

## 1 INTRODUCTION

Immune thrombocytopenic purpura (ITP) is an autoimmune hematological disorder where antibodies directed against platelets lead to premature platelet destruction, resulting in low platelet count and increased tendency for mucocutaneous bleeding. It is a relatively rare disease with an incidence of about 10‐125 cases per million per year.^1^

Despite thrombocytopenia, there is an increased risk of thrombotic complications due to the presence of abnormally enlarged immature platelets and increased antibody‐mediated damage to the endothelium.^2^ Additionally, the thrombotic risk is amplified by therapies targeting ITP such as steroids, intravenous immune globulins and rituximab.^3^

Patients with ITP are at increased risk of bleeding complications and as such, those presenting with acute myocardial infarction (AMI) requiring revascularization or antiplatelet therapy may be challenging to manage.^4^, ^5^ The exact incidence of AMI in ITP is not reported in the literature and the topic appears to be mostly addressed in brief reports.^6^, ^7^, ^8^, ^9^ Moreover, guidelines on the management of AMI in ITP patients are ambiguous as ITP patients were excluded from the majority of trials addressing therapy of AMI.^6^, ^10^

Given the low incidence of ITP in the general population, there are no large center studies focusing on the impact of ITP on the outcomes of myocardial ischemia. Herein, we resort to the US‐based national level inpatient data to study the impact of ITP in patients admitted with AMI on mortality, coronary revascularization therapy, in‐hospital complications and length of stay.

## 2 METHODS

### 2.1 Study design

The study data was retrieved from the national inpatient sample (NIS), the largest all‐payer inpatient care database in the United States. The NIS contains discharge‐level data on more than seven million hospitalizations and approximates a sample of 20% of all US community hospitals. The sampling methodology adopted by the NIS allows the approximation of national estimates by applying weighting variables to the discharges.^11^ Hospitalizations within the database provide basic demographic information such as age, gender, and race as well as the international classification of diseases, ninth revision, clinical modification (ICD‐9‐CM) coded diagnoses, outcomes, total costs and lengths of hospital stay.^11^ Since we used a public database with no reports of patient personal identifying information, this study was deemed exempt by the Institutional Review Board.

### 2.2 Study population

**Figure 1:**
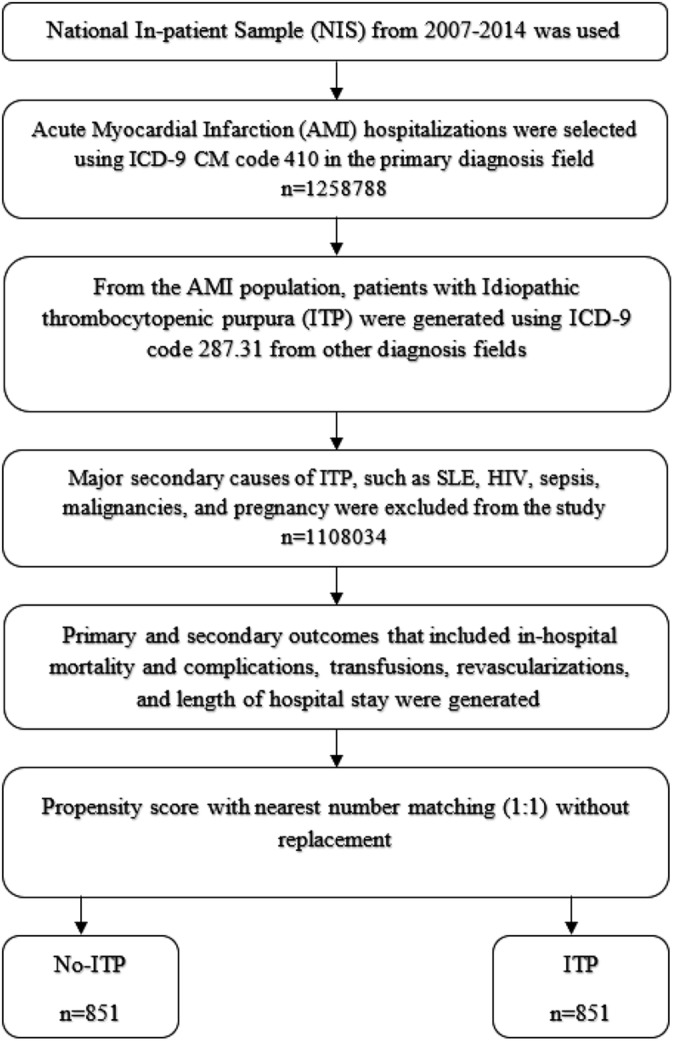
Flow chart of the study design and cohort selection. Sample size presented. Presence of the international classification of diseases, ninth revision, clinical modification (ICD‐9‐CM) codes 287.31 in the secondary diagnosis fields for ITP and clinical classifications software (ICD‐CCS) 100 for AMI in the primary diagnosis field. AMI, acute myocardial infarction; ITP, immune thrombocytopenic purpura

Our study population includes patients ≥18 years of age, admitted with a primary discharge diagnosis of AMI using the ICD‐9‐CM code 410 between 2007 and 2014, with or without ITP. A systematic review of the discharge diagnosis codes for AMI found that the ICD‐9‐CM code 410 had a 94% sensitivity and 99% specificity.^12^ ITP was identified using the ICD‐9 code 287.31 as a secondary diagnosis. Specific details of the study population are highlighted in Figure 1. Those who were discharged on the same day, transferred between facilities, or with an elective admission, were excluded from the analysis. Those hospitalized with possible secondary causes of ITP such as coagulation disorders and other causes of thrombocytopenia, such as systemic lupus erythematosus, human immunodeficiency virus (HIV), sepsis, malignancies, disseminated intravascular coagulation (DIC), thrombotic thrombocytopenic purpura (TTP), and pregnancy were excluded, as highlighted in the study by Danese et al.^13^ The ICD‐9 codes used to identify these conditions are listed in the supplementary file.

### 2.3 Outcomes

The primary outcome of interest was in‐hospital mortality. Secondary outcomes were revascularization therapies (percutaneous coronary intervention, bare metal stents, and drug eluting stents coronary bypass), bleeding (epistaxis, hematoma, gastrointestinal, genitourinary and intracranial bleeds), cardiovascular complications (cardiogenic shock, complete heart block, hemopericardium and cardiac tamponade, and iatrogenic cardiac complications), blood product transfusions and length of hospital stay. We further examined the outcomes in ITP and non‐ITP hospitalization in the following prespecified subgroups: patients with ST‐elevation myocardial infarction (STEMI) and patients with non‐ST‐elevation myocardial infarction (NSTEMI). Data pertaining to the outcomes were extracted from the NIS database using their corresponding ICD‐9 codes illustrated in the supplementary index.

### 2.4 Statistical analysis

A propensity score matching model was developed to derive two matched groups for comparative outcome analysis, to account for potential confounding factors and reduce the effect of selection bias. We used a multivariable logistic regression model with AMI with ITP as the outcome variable, and all co‐morbidities in Table 1 and patient‐level NIS weights as covariates. We used a one‐to‐one greedy matching protocol and a caliper width of 0.1 multiplied by the SD of the logit of the propensity score to create a matched cohort that includes matched demographics, comorbidities, and year of admission, between ITP and non‐ITP hospitalization with AMI.^14^, ^15^ We first conducted bivariate analyses to compare demographic, clinical and hospital characteristics in AMI admissions with and without ITP. Chi‐square tests were used for categorical variables and the *t*‐test for continuous variables with normal distribution. For matched variables, the mean and SD were reported for continuous variables, and percentages were reported for categorical variables. Binary outcomes were modeled with binomial logistic regressions, while discrete numeric variables were modeled with generalized linear model regressions. Subsequently, a stratified analysis was done on those hospitalized with ITP who received transfusions to identify the effect of transfusion on major outcomes in ITP hospitalization. Multivariable logistic regression was performed to identify the predictors of these major outcomes. Variables included in the model were age and sex, in addition to statistically and clinically significant variables derived from univariate analysis. Cumulative in‐hospital mortality among AMI hospitalization with and without ITP was characterized using a Kaplan‐Meier plot, with the log‐rank (Mantel‐Cox) test used for comparison between the two groups.

**Table 1:** Baseline characteristics of acute myocardial infarction hospitalizations with and without immune thrombocytopenic purpura (ITP)

	Acute myocardial infarction (unmatched)			Acute myocardial infarction (matched)		
	No ITP	ITP	*P*‐value	No ITP	ITP	*P*‐value
No. of observations	1 107 021	1002		851	851	
Demographics						
Age, mean (SD) (years)	66.71 ± 14.48	71.11 ± 13.34	<.001	72.05 ± 13.39	71.45 ± 13.26	.65
Male—no. (%)	671 701 (60.7)	585 (58.4)	.14	501 (58.9)	496 (58.3)	.81
Race—no. (%)						
White	704 584 (63.6)	694 (69.3)	.001	564 (66.3)	590 (69.3)	.06
Black	90 544 (8.2)	53 (5.3)		75 (8.8)	47 (5.5)	
Hispanic	70 549 (6.4)	53 (5.3)		63 (7.4)	49 (5.8)	
Asians	20 829 (1.9)	27 (2.7)		20 (2.4)	20 (2.4)	
CVD Risk Factors—no. (%)						
Alcohol abuse	32 951 (3)	27 (2.7)	.6	25 (2.9)	23 (2.7)	.77
Smoking	395 175 (35.7)	284 (28.7)	<.001	229 (26.9)	244 (28.7)	.42
Obesity	137 276 (12.4)	107 (10.7)	.09	93 (10.9)	93 (10.9)	1
Hypertension	741 137 (66.9)	679 (67.8)	.58	601 (70.6)	582 (68.4)	.32
Dyslipidemia	624 713 (56.4)	524 (52.3)	.008	447 (52.5)	459 (53.9)	.56
Diabetes	374 859 (339)	379 (36.9)	.04	338 (39.7)	319 (37.5)	.34
Comorbidities—no. (%)						
Hypothyroidism	104 109 (9.4)	124 (12.4)	.001	125 (14.7)	114 (13.4)	.44
Congestive heart failure	1 098 579 (0.8)	992 (1)	.39	11 (1.3)	8 (0.9)	.49
Valvular disease	2491 (0.2)	4 (0.4)	.25	5 (0.6)	3 (0.4)	.48
Atrial Fibrillation	175 404 (15.8)	232 (23.2)	<.001	196 (23)	205 (24.1)	.61
Previous history of myocardial infarction	113 246 (10.2)	128 (12.8)	.008	112 (13.2)	113 (13.3)	.94
Previous history of percutaneous coronary intervention	131 438 (11.9)	124 (12.4)	.62	114 (13.4)	113 (13.3)	.94
Previous history of coronary bypass	80 916 (7.3)	110 (11)	<.001	100 (11.8)	97 (11.4)	.82
Previous history of pacemaker	27 166 (2.5)	34 (3.4)	.055	22 (2.6)	32 (3.8)	.17
Carotid artery disease	19 665 (1.8)	26 (2.6)	.05	28 (3.3)	23 (2.7)	.48
Peripheral vascular disease	116 908 (10.6)	136 (13.6)	.002	107 (12.6)	123 (14.5)	.26
Previous history of cerebrovascular disease	65 360 (5.9)	67 (6.7)	.29	60 (7.1)	62 (7.3)	.85
Chronic pulmonary disease	222 643 (20.1)	218 (21.8)	19	175 (20.6)	183 (21.5)	.63
Liver disease	12 414 (1.1)	54 (5.4)	<.001	44 (5.2)	50 (5.9)	.52
Renal Failure	171 980 (15.5)	244 (24.4)	<.001	230 (27)	216 (25.4)	.44
Maintenance Dialysis	29 435 (2.7)	51 (5.1)	<.001	48 (5.6)	46 (5.4)	.83
Fluid and electrolyte disorders	203 719 (18.4)	242 (24.2)	<.001	201 (23.6)	211 (24.8)	.57

Data extraction and analyses were performed with IBM SPSS statistics (Version 25.0 Armonk, NY). All statistical tests were two‐sided and a *P* value of <.05 was considered statistically significant.

## 3 RESULTS

### 3.1 Baseline characteristics and matched cohort

There were 1 258 788 hospitalizations with AMI between 2007 and 2014. After excluding patients with secondary causes of ITP, 1108023 hospitalization with AMI were collected and included in the study for analysis. When compared to those without ITP, those hospitalized with ITP were older, more likely to be smokers and had more comorbidities such as hypothyroidism, atrial fibrillation, previous history of liver disease and end stage renal disease and previous history of cardiovascular myocardial infarctions, coronary artery bypass surgeries, and peripheral vascular diseases (Table 1). After applying propensity matching, we obtained a sample of 1702 patients (851 in each group) with equally matched baseline characteristics (Table 1).

### 3.2 Outcomes

**Figure 2:**
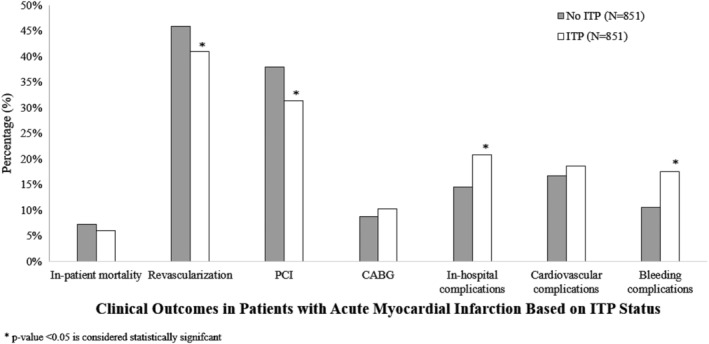
Bar graph presentation illustrating in‐hospital outcomes in AMI hospitalizations with and without ITP. AMI, acute myocardial infarction; ITP, immune thrombocytopenic purpura

Figure 2 summarizes the impact of ITP on the major in‐patient clinical outcomes of patients with AMI.

### 3.3 In‐patient short‐term mortality

There was no significant difference between patient with AMI and ITP when compared to those without ITP (6% vs 7.3%, OR:0.81; 95% CI: 0.55‐1.19; *P* = .3). This is further illustrated in Kaplan‐Meier curves (Figure S1) showing no difference in cumulative survival between hospitalizations for AMI of patients with and without ITP at different time intervals since admission (*P* = .5 using log‐rank test). When stratified based on the type of AMI (STEMI or NSTEMI), it was found that there was no difference in short‐term inpatient mortality between hospitalizations of ITP and non‐ITP patients for both STEMI (8.6% vs 14.9%, OR:0.54; 95%CI: 0.28‐1.00; *P* = .05) and NSTEMI (5.3% vs 4.6%, OR:1.15; 95%CI: 0.70‐1.91; *P* = .6) (Table 2).

**Table 2:** Clinical outcomes of hospitalizations with and without ITP among AMI, STEMI, and NSTEMI

Patient sample	AMI (N = 1702)				STEMI (N = 409)				NSTEMI (N = 1293)			
	No ITP (N = 851)	ITP (N = 851)	OR (95%CI)	*P*‐value	No ITP (N = 222)	ITP (N = 187)	OR (95%CI)	*P*‐value	No ITP (N = 629)	ITP (N = 664)	OR (95%CI)	*P*‐value
In‐patient mortality	62 (7.3)	51 (6)	0.81 (0.55‐1.19)	.3	33 (14.9)	16 (8.6)	0.54 (0.28‐1.00)	.05	29 (4.6)	35 (5.3)	1.15 (0.70‐1.91)	.6
Revascularization	391 (45.9)	348 (40.9)	0.81 (0.67‐0.98)	.03	136 (61.3)	124 (66.3)	1.24 (0.83‐1.87)	.3	255 (40.5)	224 (33.7)	0.75 (0.59‐0.94)	.01
Percutaneous coronary intervention	323 (38)	266 (31.3)	0.74 (0.61‐0.91)	.004	126 (56.8)	112 (59.9)	1.14 (0.77‐1.69)	.5	197 (31.3)	154 (23.2)	0.66 (0.52‐0.85)	.001
Bare metal stents	96 (11.3)	131 (15.4)	1.43 (1.08‐1.90)	.01	38 (17.1)	58 (31)	2.2 (1.37‐3.47)	.0001	58 (9.2)	73 (11)	1.22 (0.85‐1.75)	.3
Drug eluting stents	209 (24.6)	108 (12.7)	0.45 (0.35‐0.58)	<.0001	74 (33.3)	43 (23)	0.59 (0.38‐0.93)	.02	135 (21.5)	65 (9.8)	0.39 (0.29‐0.55)	<.0001
Coronary bypass	75 (8.8)	88 (10.3)	1.19 (0.86‐1.65)	.3	14 (6.3)	15 (8)	1.3 (0.61‐2.76)	.5	61 (9.7)	73 (11)	1.15 (0.80‐1.65)	.45
In‐hospital complications	123 (14.5)	177 (20.8)	1.55 (1.21‐2.00)	.001	27 (12.2)	38 (20.3)	1.84 (1.08‐3.15)	.025	96 (15.3)	139 (20.9)	1.47 (1.10‐1.96)	.008
Use of mechanical circulatory support	44 (5.2)	33 (3.9)	0.7 (0.47‐1.17)	.2	27 (12.2)	17 (9.1)	0.72 (0.38‐1.37)	.3	17 (2.7)	16 (2.4)	0.9 (0.44‐1.78)	.7
Cardiovascular complications^a^	142 (16.7)	158 (18.6)	1.14 (0.89‐1.46)	.3	36 (16.2)	34 (18.2)	1.15 (0.69‐1.92)	.6	106 (16.9)	124 (18.7)	1.13 (0.85‐1.51)	.4
Cardiac complications^b^	43 (5.1)	44 (5.2)	1.03 (0.66‐1.58)	.9	15 (6.8)	12 (6.4)	0.95 (0.43‐2.07)	.9	28 (4.5)	32 (4.8)	1.1 (0.65‐1.83)	.7
VTE	106 (12.5)	120 (14.1)	1.15 (0.87‐1.53)	.3	24 (10.8)	23 (12.3)	1.16 (0.63‐2.13)	.6	82 (13)	97 (14.6)	1.14 (0.83‐1.57)	.4
Acute ischemic stroke	39 (4.6)	40 (4.7)	1.03 (0.65‐1.61)	.9	10 (4.5)	6 (3.2)	0.7 (0.25‐1.97)	.5	29 (4.6)	34 (5.1)	1.1 (0.67‐1.86)	.7
Bleeding complications^c^	90 (10.6)	149 (17.5)	1.80 (1.36‐2.38)	<.0001	13 (5.9)	31 (16.6)	3.20 (1.62‐6.31)	<.0001	77 (12.2)	118 (17.8)	1.55 (1.14‐2.11)	.005
Transfusions^d^	89 (10.5)	143 (16.8)	1.73 (1.30‐2.30)	<.0001	13 (5.9)	31 (16.6)	3.20 (1.62‐6.31)	<.001	76 (12.1)	112 (16.9)	1.48 (1.08‐2.02)	.015
Platelet transfusion	7 (0.8)	56 (6.6)	8.5 (3.85‐18.75)	<.0001	5 (0.8)	43 (6.5)	8.6 (3.4‐21.96)	<.001	2 (0.9)	13 (7)	8.22 (1.8‐36.9	.001
Length of stay average(d)	5.40	6.14	1.14 (1.05, 1.23)	.002	5.02	5.09	1.01 (0.86; 1.20)	.9	5.54	6.44	1.16 (1.06, 1.27)	.001

Abbreviations: AMI, acute myocardial infarction; ITP, immune thrombocytopenic purpura; STEMI, non‐ST‐elevation myocardial infarction; STEMI, ST‐elevation myocardial infarction; VTE, venous thromboembolic events.

a Cardiovascular complications included cardiac complications, VTE, and acute ischemic stroke.

b Cardiac complications include cardiogenic shock, complete heart block, pericardial complications such as hemopericardium and cardiac tamponade, and iatrogenic cardiac complications.

c Bleeding complications includes gastrointestinal, genitourinary, and intracranial bleeding, hematomas, epistaxis, and bleeding requiring transfusions.

d Transfusions included patients that either received blood or platelet products.

### 3.4 Revascularization therapy

Compared to those without ITP, those hospitalized with AMI and ITP had fewer coronary revascularizations (40.9% vs 45.9%, OR: 0.81; 95%CI: 0.67‐0.98; *P* = .03), with less percutaneous coronary interventions (PCI) (31.3% vs 38%, OR: 0.74; CI: 0.61‐0.91; *P* = .004) and more coronary artery bypass grafting (CABG) (10.3% vs 8.8%, OR: 1.19; 95%CI: 0.861.65; *P* = .3). When analyzing hospitalizations for STEMI and NSTEMI, those who were admitted with ITP and STEMI had similar rates of revascularization with either PCI or CABG compared to non‐ITP (Table 2). However, those admitted with NSTEMI and ITP were less likely to undergo PCI (23.2% vs 31.3%, OR: 0.66; 95%CI: 0.52‐0.85; *P* = .001) compared to those hospitalized with no ITP, but no difference was noted using CABG as a method of revascularization.

### 3.5 Implantation of bare metal vs drug eluting stent

Among patients admitted with AMI who had PCI, bare metal stents were used more in patients with ITP (15.4% vs11.3%, OR:1.43;95% CI:1.08‐1.90; *P* = .01) whereas drug eluting stents were used more in patient with non‐ITP (12.7% vs 24.6%, OR = 0.45; 95% CI:0.35‐0.58; *P* < .0001). The proportion of ITP vs non‐ITP patients among those who obtained a bare metal stent and those who obtained a drug eluting stent were 55% vs 45% and 32.3% vs 67.7%, respectively (*P* < .0001). Similar findings were noted in those admitted with STEMI and ITP, in which there was a greater tendency to use of bare metal stents and lesser utilization of drug eluting stents relative to those hospitalized without ITP. On the other hand, no difference was noted in the use of bare metal stents between ITP and non‐ITP admissions with NSTEMI, although there was a lesser tendency to use drug eluting stents in those admitted with ITP.

### 3.6 In‐hospital complications

Overall, hospitalizations with ITP had a higher risk of in‐hospital complications (OR: 1.55; CI: 1.21‐2.00; *P* = .001). Similar rates were observed when stratified based on STEMI (OR: 1.84; 95% CI: 1.08‐3.15; *P* = .025) and NSTEMI (OR: 1.47; 95% CI:1.10‐1.96; *P* = .008).

#### 3.6.1 Cardiovascular complications

Cardiovascular complications included cardiac complications (cardiogenic shock, complete heart block, pericardial complications such as hemopericardium and cardiac tamponade, and iatrogenic cardiac complications), venous thromboembolic events (VTE), and acute ischemic stroke. Similar rates of cardiac, VTE, and acute ischemic strokes were noted among all patients admitted with AMI, STEMI and NSTEMI, irrespective of their ITP status (Table 2).

#### 3.6.2 Bleeding complications

Bleeding complications were defined as any reports of gastrointestinal, genitourinary, and intracranial bleeding, hematomas, epistaxis, and bleeding requiring transfusions. Relative to those with no history of ITP, hospitalizations with AMI and ITP had increased risk of bleeding complications (OR:1.80; CI: 1.36‐2.38; *P* < .0001) and increased transfusions (OR: 1.73; CI: 1.30‐2.30; *P* < .0001). In addition, those admitted with ITP and AMI were more likely to require platelet transfusions compared to non‐ITP (OR: 8.50; CI: 3.85‐18.75; *P* < .0001). Similar findings were noted when stratified by STEMI and NSTEMI (Table 2).

### 3.7 Length of hospital stay

Overall, hospitalizations with ITP had a longer hospital stay compared to non‐ITP (6.14 vs 5.4 days; mean ratio: 1.14; CI: 1.05‐1.23; *P* = .002) with AMI. When stratified by STEMI and NSTEMI, those with ITP and NSTEMI had a significantly longer mean hospital stay compared to non‐ITP admissions (6.44 vs 5.54 days, *P* = .001), but no difference was noted among STEMI hospitalizations (5.09 vs 5.02 days, *P* = .9).

### 3.8 Transfusion of blood products

We also explored whether transfusion of blood products (including both platelets and packed red blood cells) were predictors of major adverse events in those admitted with ITP (Table S1). After adjusting for demographic variables and cardiovascular risk comorbidities, those who received transfusions had similar risk of death (OR:1.21; CI: 0.6‐2.46; *P* = .6), but a higher risk of acute ischemic stroke (OR:4.52; CI: 2.35‐8.69; *P* < .0001), VTE (OR:2.62; CI: 1.51‐4.53; *P* = .001), and cardiac complications (OR:3.06; CI: 1.61‐5.82; *P* < .0001) when compared to those who did not receive transfusions. Hospitalizations with ITP and AMI were then stratified based on whether they either received platelet transfusions or packed red blood cells alone. Among those who only received either platelet transfusions or packed red blood cell transfusions, mortality in either group was not found to be significant (Table S1). Those who received platelet transfusions were highly associated with developing an acute ischemic stroke (OR:5.54; CI: 2.55‐12.03; *P* < .0001), VTE (OR:3.80; CI:1.88‐7.68; *P* < .0001), and cardiac complications (OR:2.93; CI:1.24‐6.90; *P* = .01) when compared to those who did not receive platelet transfusions. In addition, among those who received packed red blood cell transfusions, there was a greater association with developing an acute ischemic stroke (OR:2.41; CI: 1.07‐5.43; *P* = .03) and cardiac complications (OR:2.40; CI:1.11‐5.18; *P* = .02) but no difference in VTE after adjustment (OR:1.54; CI:0.75‐3.13; *P* = .2).

## 4 DISCUSSION

The findings from this NIS‐based propensity matched cohort study over the period 2007‐2014, can be summarized as follows: patients with ITP and AMI were less likely to undergo revascularization, had more bleeding complications and a higher rate of transfusions compared to non‐ITP patients with AMI. There was no difference in short‐term inpatient mortality between ITP and non‐ITP patients with AMI. However, patients with ITP had a longer hospital stay compared to non‐ITP patients.

Despite being thrombocytopenic, ITP patients experience both arterial and venous thromboembolic events. Moreover, patients with ITP have been shown to be at increased risk for developing coronary artery disease and thrombosis.^4^ The heightened risk has been attributed to the presence of larger and more adhesive platelets, release of thrombotic platelet microparticles (PMP), hypercoagulability induced by steroid and/or IVIG treatment, and antibody‐mediated attack on endothelial cells caused by cross‐reactivity.^16^, ^17^, ^18^, ^19^ In addition, comorbid conditions in ITP patients may contribute to the added risk. In our study, ITP was associated with a significantly increased risk of diabetes, liver disease, renal failure, peripheral vascular disease, and previous history of myocardial infarction. On the other hand, ITP is associated with an increased risk of bleeding, which poses a challenge for clinicians in the management of ITP patients presenting with AMI. In this study, we observed a lower rate of revascularization in ITP patients compared to non‐ITP patients with AMI. This difference was due to lower rates of PCI in ITP patients, as there was no significant difference in the rates of CABG between the two groups. Specifically, the rate of PCI was similar in patients with STEMI, but lower in patients with NSTEMI. This finding can be potentially explained by a decreased tendency for clinicians to perform PCI in ITP patients with non‐emergent indications. Several studies also evaluated the trend in revascularization in patients with AMI, and similar findings were observed. A study by Kolte et al. (2016) highlighted, “revascularization rates were significantly higher in patients with cardiogenic shock versus without cardiogenic shock (72.5% vs. 62.6%, p <0.001) among patients who underwent coronary angiography.”^20^ However, in another study by Khera et al. (2015), revascularization rates were similar such that PCI was performed in 68.4% of women and 76.7% of men who experienced STEMI. This latter study primarily looked into patients ranging from 18 to 59 years old.^21^ Thus, our data reflects a real‐world situation and the population which is much more heterogeneous compared to myocardial infarction‐research populations. Moreover, our study found that there is greater tendency to place bare metal stents and lesser tendency to place drug eluting stents in ITP patients with AMI compared to the non‐ITP patients. These findings were consistent with Ayoub et al, in which they found that patients with a history of chronic thrombocytopenia are more likely to undergo bare metal stentings and less likely to undergo drug eluting stents compared to patients with normal platelet levels.^22^ It is likely that physicians prefer the use of bare metal stents in patients with lower platelet levels to avoid longer treatment with dual anti‐platelet therapy as highlighted in a review by McCarthy et al.^23^ However, two new randomized controlled trials have demonstrated the safety and superiority of the use of second‐generation drug‐eluting stents compared to bare metal stents in patients with thrombocytopenia during an AMI.^23^, ^24^, ^25^ Currently, there is still limited evidence to guide management of ITP patients along other thrombocytopenic patients who develop AMI, including the preferred revascularization method, and the use of antiplatelets and anticoagulation.^23^, ^26^, ^27^ In addition, major trials such as the TRITON‐TIMI 38, PLATO, CHAMPION, PHOENIX, and CURE have excluded patients with thrombocytopenia.^23^, ^28^, ^29^, ^30^, ^31^ In a report by Russo et al, both PCI and CABG were successfully performed in patients with ITP (32 patients with CABG and 15 patients with PCI). However, this was associated with increased bleeding risk compared to the general population. The rate of significant bleeding was 12.5% and 6% in the CABG and PCI groups, respectively.^32^ In our study, there was a significantly increased risk of bleeding complications in patients with ITP and STEMI but not in ITP patients with NSTEMI. This may be explained by the higher rate of PCI and stenting in the group with STEMI which requires a more intensive antiplatelet regimen. Despite the higher rate of bleeding complications, there was no significant difference in inpatient mortality between the two groups, which may indicate that bleeding complications were not fatal. However, this only describes in‐patient/short‐term mortality. The impact of thrombocytopenia on prognosis after PCI has been studied by several groups, with conflicting results. In an NIS‐based study by Ayoub et al., PCI in patients with chronic thrombocytopenia was associated with an increased risk of bleeding complications, transfusions, vascular complications, ischemic but not hemorrhagic CVA, and in‐hospital mortality. However, the increased mortality was not explained by increased bleeding complications alone.^22^ In a pooled analysis of patients enrolled in two major clinical trials (ACUITY and HORIZONS‐AMI), Yadaz et al. found that baseline thrombocytopenia was an independent predictor for major cardiac events and all‐cause mortality at 1 year in ACS patients who underwent PCI.^33^ A pooled analysis of three large Japanese studies by Ito et al, showed that thrombocytopenia was associated with an increase of major bleeding events and all‐cause mortality during the entire 3‐year follow up period.^34^ On the other hand, a single center retrospective study at Mayo Clinic showed no significant difference in inpatient bleeding and inpatient deaths after PCI in patients with thrombocytopenia and a matched control group, but a significantly higher rate of transfusion in the thrombocytopenia group. Interestingly, there was an increase rate of long‐term bleeding in thrombocytopenia patients, which was not in excess in the first year where dual antiplatelets are used, compared to subsequent years.^35^ It is difficult to derive conclusions from these studies and extrapolate results to patients with ITP, as included patients had heterogenous causes of thrombocytopenia, where the pathophysiology, comorbidities and bleeding risks differ.

In this study, the length of hospitalization was found to be increased in ITP patients with AMI. Interestingly, this finding was seen in patients with NSTEMI, but not in patients with STEMI. A possible explanation for this is that STEMI patients require urgent revascularization compared to NSTEMI. A cross sectional study by Sugiyama et al, found that patients that underwent urgent PCI had shorter length of hospital stay.^36^ In addition, several other studies found that there is increased length of hospital stay with NSTEMI patients which require optimal risk stratification and medical management.^37^, ^38^ Limited data exist on length of hospital stay in ITP patients but according to Ayoub et al., patients with chronic thrombocytopenia who were admitted for PCI had a significantly longer hospital stay compared to those without thrombocytopenia.^22^ This was consistent with our results in ITP patients as bleeding complications and transfusions were higher than the general population and, as such, may contribute to increase length of hospital stay.

The increased need in transfusion rate observed in our study was consistent with findings by Ayoub et al. and Raphael et al. in patients with ACS and thrombocytopenia.^22^, ^35^ Although platelet transfusions have been associated with an increased risk of arterial thrombosis and mortality in patients with thrombotic thrombocytopenic purpura and heparin‐induced thrombocytopenia, this finding has not been observed in patients with ITP.^39^ In a large meta‐analysis published on the effect of blood transfusions on patients with AMI, there was a 12% increased risk of mortality in these patients independent of hemoglobin level.^40^ On the other hand, a retrospective study on elderly patients with AMI showed an increased 1‐year mortality in those who received blood when hemoglobin was >10 g/dL but a 50% reduction in 1‐year mortality when hemoglobin was <8 g/dL.^41^ In our study, there was no difference in mortality among ITP patients admitted for AMI who had received either blood or platelet transfusions. Yet those patients had a significantly higher number of cardiovascular complications. It is not possible to determine if these complications preceded the transfusions. Patients with indications for blood product transfusion are generally at an advanced disease state and at risk for cardiovascular complications.^42^ To our knowledge, our study is the first to report an association between blood products transfusion in patients with ITP admitted for AMI and cardiovascular complications. This signifies future studies to look at transfusion and hemoglobin levels as part of a risk model in predicting cardiovascular outcomes.

Several limitations to this study need to be acknowledged. The NIS is a de‐identified administrative database and thus, it would not be possible to validate individual ICD‐9 codes. This could potentially have impacted our analysis, nevertheless, the same ICD‐9 codes were used through the entire study period. Furthermore, this database does not provide information on the severity of thrombocytopenia as this may have had an important prognostic effect on the study outcomes. In addition, no data on therapy (anticoagulation, antiplatelets, etc.) was available. Also, no reports were available for vascular access during PCI as undergoing coronary catheterization during femoral artery is associated with higher risk of bleeding complications.^43^ Since the study is cross sectional in the nature, establishment of causality was not possible.

Reverse causality is however less likely as the studied outcomes are not known to cause ITP. Finally, since the database relies heavily on reported diagnoses, our study is at high risk for misclassification bias. Misclassification in this case is more likely to be nondifferential, drawing the study results toward the null hypothesis. These limitations were compounded by a large sample size of an underrepresented population along with the use of propensity‐matched cohorts which significantly reduced the risk of both selection and confounding bias.

## 5 CONCLUSION

Despite no difference in in‐hospital mortality between ITP and non‐ITP patients admitted for AMI, those with ITP were more likely to have bleeding complications and require a longer hospital stay. Bare metal stents were more likely to be utilized in ITP patients than drug eluting stents. Moreover, ITP patients who required transfusions were found to have a higher frequency of cardiovascular complications. This reflects the need for evidence‐based guidelines to standardize the management of patients with ITP and other thrombocytopenic disorders presenting with AMI, assess the best revascularization strategy, platelet cutoff for interventions, antiplatelet choice and duration, and perioperative strategies to minimize complications and improve outcomes in this under‐represented population.

## CONFLICT OF INTEREST

The authors declare no potential conflict of interests.

## Supporting information


**Figure S1** Comparison of in‐hospital mortality in AMI hospitalizations with and without ITP. Kaplan‐Meier curves showing difference between cumulative in‐hospital survival for both groups at different time interval since admission (*P* = 0.5 using log‐rank test).

**Supplementary Table 1:** Patients with ITP admitted with AMI stratified based by transfusion status
**Supplementary Table 2:** Diagnosis Codes
**Supplementary Table 3:** ICD‐9 codes for in‐hospital outcomes
