# Supplementary figures and images for: Impact of immune thrombocytopenic purpura on clinical outcomes in patients with acute myocardial infarction
Source: Clin Cardiol. 2019 Nov 11;43(1):50–9. doi: 10.1002/clc.23287 (PMC6954382; doi:10.1002/clc.23287)

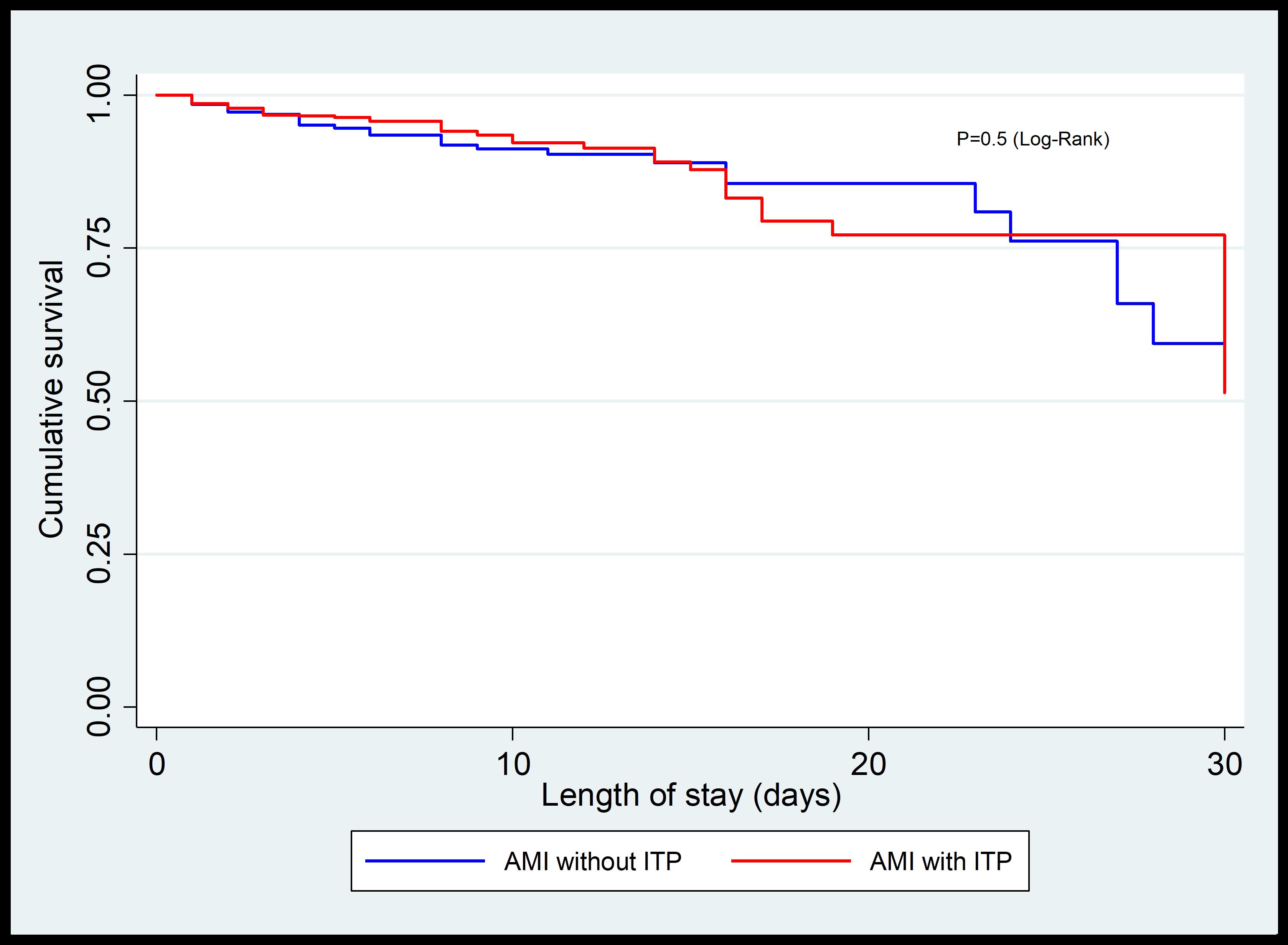

Supplement: Supplementary file 1 — Figure S1 Comparison of in‐hospital mortality in AMI hospitalizations with and without ITP. Kaplan‐Meier curves showing difference between cumulative in‐hospital survival for both groups at different time interval since admission (P = 0.5 using log‐rank test). [file CLC-43-50-s001.jpg]
