# Supplementary material for: Impact of immune thrombocytopenic purpura on clinical outcomes in patients with acute myocardial infarction
Source: Clin Cardiol. 2019 Nov 11;43(1):50–9. doi: 10.1002/clc.23287 (PMC6954382; doi:10.1002/clc.23287)
Supplement: Supplementary file 2 — Supplementary Table 1: Patients with ITP admitted with AMI stratified based by transfusion status Supplementary Table 2: Diagnosis Codes Supplementary Table 3: ICD‐9 codes for in‐hospital outcomes [file CLC-43-50-s002.docx]

SUPPLEMENTARY MATERIAL

| **Supplementary Table1 : Patients with ITP admitted with AMI stratified based by transfusion status** | | | | | | |
| --- | --- | --- | --- | --- | --- | --- |
| **Overall Transfusion** | | | | | | |
| **In-Hospital Outcomes** | **Transfusion**  **(N=143)** | **No Transfusion**  **(N=708)** | **Unadjusted Odds Ratio (95% CI)** | **p-value** | **Adjusted Odds Ratio (95% CI)** | **p-value** |
| Mortality | 12 (8.4) | 39 (5.5) | 1.57 (0.80-3.08) | 0.2 | 1.21 (0.60-2.46) | 0.6 |
| Acute ischemic stroke | 18 (12.6) | 22 (3.1) | 4.49 (2.34-8.61) | <0.0001 | 4.52 (2.35-8.69) | <0.0001 |
| VTE | 28 (19.6) | 92 (13) | 1.63 (1.02-2.60) | 0.04 | 2.62 (1.51-4.53) | 0.001 |
| Cardiac complications | 16 (11.2) | 28 (4) | 3.06 (1.61-5.82) | <0.0001 | 3.06 (1.61-5.82) | 0.001 |
| **Platelet Transfusion** | | | | | | |
| **In-Hospital Outcomes** | **Transfusion**  **(N=56)** | **No Transfusion**  **(N=795)** | **Unadjusted Odds Ratio (95% CI)** | **p-value** | **Adjusted Odds Ratio (95% CI)** | **p-value** |
| Mortality | 6 (10.7) | 45 (5.7) | 2.00 (0.81-4.91) | 0.1 | 1.64 (0.62-4.31) | 0.3 |
| Acute ischemic stroke | 10 (17.9) | 30 (3.8) | 5.54 (2.55-12.03) | <0.0001 | 5.54 (2.55-12.03) | <0.0001 |
| VTE | 13 (23.2) | 107 (13.5) | 1.94 (1.01-3.73) | 0.04 | 3.80 (1.88-7.68) | <0.0001 |
| Cardiac complications | 7 (12.5) | 37 (4.7) | 2.93 (1.24-6.90) | 0.01 | 2.93 (1.24-6.90) | 0.01 |
| **Red Blood Cell Transfusion** | | | | | | |
| **In-Hospital Outcomes** | **Transfusion**  **(N=87)** | **No Transfusion**  **(N=764)** | **Unadjusted Odds Ratio (95% CI)** | **p-value** | **Adjusted Odds Ratio (95% CI)** | **p-value** |
| Mortality | 6 (6.9) | 45 (5.9) | 1.18 (0.49-2.86) | 0.7 | 0.9 (0.36-2-2.24) | 0.8 |
| Acute ischemic stroke | 8 (9.2) | 32 (4.2) | 2.32 (1.03-5.20) | 0.04 | 2.41 (1.07-5.43) | 0.03 |
| VTE | 15 (17.2) | 105 (13.7) | 1.31 (0.72-2.37) | 0.4 | 1.54 (0.75-3.13) | 0.2 |
| Cardiac complications | 9 (10.3) | 35 (4.6) | 2.40 (1.11-5.18) | 0.02 | 2.40 (1.11-5.18) | 0.02 |
| Variables included in the model: age, gender, obesity, history of diabetes mellitus, hypertension, dyslipidemia, chronic kidney disease, chronic pulmonary disease, liver failure, congestive heart failure, history of previous myocardial infarction or stroke. | | | | | | |

Baseline comorbidities were identified using Agency of Healthcare and Qaulity Research ( AHRQ ) tool :

[**https://www.hcup-us.ahrq.gov/db/nation/nis/nisdbdocumentation.jsp**](https://www.hcup-us.ahrq.gov/db/nation/nis/nisdbdocumentation.jsp)

**Supplementary Table 2:** **Diagnosis Codes**

| **Diagnosis** | **ICD-9 Codes** |
| --- | --- |
| Immune Thrombocytopenic Purpura | 287.31 |
| History of stroke | V12.54, 438.x |
| Atrial Fibrillation | 427.31 |
| Prior PCI | V45.82 |
| History of CABG | V45.81 |
| Prior Pacemaker Implantation | V45.01 |
| STEMI | 410.0x, 410.1x, 410.2x, 410.3x, 410.4x, 410.5x, 410.6x, 410.8x, 410.9x |
| NSTEMI | 410.7 |
| Dyslipidemia | CCS: 53 |
| History of carotid artery disease | 433.10 |

**Supplementary Table 3: ICD-9 codes for in-hospital outcomes**

| **Any procedural complications** | **ICD CODE** |
| --- | --- |
| PCI | 00.66, 36.06, 36.07, 17.55 |
| CABG | 36.1x |
| Bare metal Stents | 36.06 |
| Drug eluting stents | 36.07 |
| Use of Mechanical Support  Intra-Aortic Balloon Pump  Percutaneous Vascular Assist Device  Extracorporeal membrane oxygenation | 3761  3768, 3762  3965 |
| Vascular complications* | 99.00-99.09, V58.2, E873.0, E934.7, E876.0, 998.11, 998.12, 34.09, 21.01-21.09, 42.33, 45.43, 44.44, 54.19, 39.41, 60.94, 6.02, 34.03, 54.12, 49.95, 57.93 |
| Gastrointestinal bleed | 456.00, 456.20, 530.70, 530.82, 531.00, 531.01, 531.20, 531.21, 531.40, 531.41, 531.60, 531.61, 532.00, 532.01, 532.20, 532.21, 532.40, 532.41, 532.60, 532.61, 533.00, 533.01, 533.20, 533.21, 533.40, 533.41, 533.60, 533.61, 534.00, 533.41, 534.20, 534.21, 534.40, 534.41, 534.60, 569.30, 578.00, 578.10, 578.90 |
| Genitourinary bleed | 599.70 626.20 626.30 626.60 626.70 626.80 627.00 627.10 |
| Acute hemorrhagic CVA | 430, 431, 432.X |
| Epistaxis | 784.7 |
| Cardiac complications† | 423x, 785.51, 426.0, |
| Platelet transfusion | 99.05 |
| DVT/PE | 415.1, 415.13, 415.19, 453.4x, 453.8x, 453.82, 453.83, 453.9 |
| Acute Ischemic Stroke | CCS: 109,110 |
| Post-procedure hemorrhage | 988.11, 998.12, 285.1, 459.0 |
| Post-procedure hemorrhage requiring transfusion | 99.00, 99.02, 99.03, 99.04, 99.08, 99.09 |
|  |  |
|  |  |
|  |  |

*****Vascular complication was defined as any transfusion procedure codes 99.00-99.09 or ICD-9 diagnosis code V58.2, E873.0, E934.7, or E876.0), any hemorrhage or hematoma (ICD-9 diagnosis code 998.11 or 998.12), or the need for percutaneous or surgical intervention to control the bleeding event (ICD-9 procedure code 34.09, 21.01-21.09, 42.33, 45.43, 44.44, 54.19, 39.41, 60.94, 6.02, 34.03, 54.12, 49.95, or 57.93)

†*Cardiac complications* include cardiogenic shock, complete heart block, pericardial complications such as hemopericardium and cardiac tamponade, and iatrogenic cardiac complications

**Figure S1.** Comparison of in-hospital mortality in AMI hospitalizations with and without ITP. Kaplan-Meier curves showing difference between cumulative in-hospital survival for both groups at different time interval since admission (p=0.5 using log-rank test).
